# T‐Cell–Redirecting Immunotherapies in Relapsed/Refractory Mantle Cell Lymphoma: Current Evidence, Sequencing, and Future Directions

**DOI:** 10.1111/ejh.70063

**Published:** 2025-11-09

**Authors:** Santino Caserta, Enrica Antonia Martino, Ernesto Vigna, Antonella Bruzzese, Nicola Amodio, Eugenio Lucia, Virginia Olivito, Caterina Labanca, Francesco Mendicino, Fortunato Morabito, Massimo Gentile

**Affiliations:** ^1^ Department of Onco‐Haematology Haematology Unit Cosenza Italy; ^2^ Department of Experimental and Clinical Medicine University of Catanzaro Catanzaro Italy; ^3^ AIL Sezione di Cosenza Cosenza Italy; ^4^ Department of Pharmacy, Health and Nutritional Science University of Calabria Rende Italy

**Keywords:** bispecific antibodies, CAR‐T therapy, immunotherapy, mantle cell lymphoma, treatment sequencing

## Abstract

Relapsed/refractory (R/R) mantle cell lymphoma (MCL) remains a therapeutic challenge, particularly in patients with high‐risk features or prior exposure to Bruton's tyrosine kinase inhibitors (BTKis). The advent of T‐cell–redirecting immunotherapies, including chimeric antigen receptor T‐cell (CAR‐T) therapy and bispecific antibodies (BsAbs), has transformed the treatment landscape. CAR‐T therapies, such as brexu‐cel and liso‐cel, induce high overall response rates and durable remissions, even in heavily pretreated or BTKi‐refractory patients. However, CAR‐T administration is limited by logistical constraints, the need for bridging therapy, and the risk of severe toxicities, including cytokine release syndrome (CRS) and immune effector cell‐associated neurotoxicity syndrome (ICANS). BsAbs, targeting CD20 and CD3, offer an off‐the‐shelf, repeatable immunotherapeutic option suitable for outpatient use, with generally manageable toxicities. Step‐up dosing, corticosteroids, and anti‐IL6 therapy mitigate CRS, while hematologic toxicity and infections require vigilant monitoring. Clinical data indicate that BsAbs are active in both CAR‐T–naïve and post‐CAR‐T settings, providing disease control in patients ineligible for immediate CAR‐T therapy. Emerging evidence supports rational sequencing and combinatorial strategies to optimize outcomes. BsAbs may be employed as a bridge to CAR‐T, or CAR‐T may be used to consolidate BsAb‐induced remissions. Combination regimens, including CAR‐T or BsAbs with BTK inhibitors or other targeted agents, are under investigation to enhance the depth and durability of response. In conclusion, CAR‐T and BsAbs are complementary modalities in R/R MCL. Individualized therapeutic sequencing and rational combinations, tailored to disease biology and patient characteristics, represent the next frontier for improving long‐term outcomes in this historically high‐risk population.

## 1 Introduction

Mantle cell lymphoma (MCL) is a rare and biologically heterogeneous subtype of non‐Hodgkin lymphoma (NHL). Its clinical spectrum ranges from indolent forms associated with long‐term survival to highly aggressive variants with dismal outcomes. At diagnosis, the majority of patients present with advanced‐stage disease and frequent extranodal involvement, including bone marrow (53%–82%), peripheral blood (≈50%), liver (≈25%), and gastrointestinal tract (20%–60%). The molecular hallmark of MCL is the *CCND1* translocation, resulting in cyclin D1 overexpression, although rearrangements of *CCND2* or *CCND3* may occasionally occur [1].

B‐cell receptor (BCR) signaling constitutes the key proliferative driver and disease heterogeneity partly reflects distinct cells of origin: naïve‐like B cells are typically aggressive, whereas memory‐like B‐cell‐derived MCL follows a more indolent course. The 2022 ICC/WHO classification distinguishes two major subtypes: nodal MCL (80%–90%), usually characterized by unmutated *IGHV*, SOX11 positivity and aggressive behavior; and non‐nodal leukemic MCL (10%–20%), typically showing mutated *IGHV*, SOX11 negativity and an indolent course. Histologically, classical, pleomorphic and blastoid variants are recognized, with pleomorphic and blastoid subtypes associated with higher proliferative activity and poor prognosis [2].

Historically considered incurable, MCL therapy was primarily aimed at symptom control and survival prolongation, particularly in the relapsed/refractory (R/R) setting.

Prognosis is largely based on clinical and biological features. The mantle cell lymphoma international prognostic index (MIPI), which incorporates age, performance status, serum LDH, and leukocyte count, stratifies patients into low‐, intermediate‐, and high‐risk groups, with significantly different survival outcomes. Additional adverse features include advanced stage, splenomegaly, extranodal involvement, B symptoms, anemia, and elevated β2‐microglobulin. Refinements such as the MIPI‐c, which integrates Ki‐67 expression, further improve risk discrimination; Ki‐67 > 30% identifies patients with markedly inferior survival. Moreover, blastoid histology, high proliferative index, *TP53* mutations, and *del(17p)* define a high‐risk biology that is resistant to intensive chemotherapy [3].

In younger, fit patients, frontline therapy traditionally consisted of intensive chemoimmunotherapy, most commonly anthracycline‐ and platinum‐based, followed by autologous stem cell transplantation (ASCT) consolidation in complete responders. Rituximab maintenance post‐ASCT has been shown to improve survival. Recently, the TRIANGLE study has reshaped the therapeutic landscape of first‐line MCL, challenging the long‐standing paradigm of ASCT as routine consolidation. By integrating BTK inhibitors into the initial treatment algorithm, the study demonstrates that durable remissions can be achieved without transplant consolidation. Additionally, early exposure to BTK inhibitors enables prompt identification of patients who are refractory or relapse rapidly, facilitating timely transition to alternative therapies or enrolment in clinical trials [4]. In the R/R setting, Bruton tyrosine kinase inhibitors (BTKi), particularly ibrutinib, have demonstrated superior efficacy compared with chemotherapy, establishing them as the preferred second‐line option.

Although novel targeted therapies and chemoimmunotherapy have markedly improved outcomes in MCL, allogeneic hematopoietic stem cell transplantation (allo‐HSCT) remains the only potentially curative option. Its use is generally limited to younger, fit patients with relapsed or refractory disease, due to the substantial risk of treatment‐related morbidity and mortality [5].

## 2 Chimeric Antigen Receptor (CAR)‐T Cell Therapy

### 2.1 Mechanism of Action

Over the last decade, CAR T‐cell therapy has revolutionized the treatment of B‐cell NHLs. Initially approved for diffuse large B‐cell lymphoma (DLBCL), its indications have since expanded to include follicular lymphoma (FL), marginal zone lymphoma, and R/R MCL [6].

Currently available products used in NHL are autologous, CD19‐directed CARs composed of an extracellular single‐chain variable fragment (scFv) through a hinge and transmembrane domain to an intracellular CD3ζ activation module, coupled with either a CD28 or 4‐1BB co‐stimulatory domain. CAR T‐cell therapy redirects. This design enables patient‐derived T cells to recognize CD19 on malignant B cells independently of human‐leucocyte‐antigen (HLA) presentation. Manufacturing involves leukapheresis, ex vivo T‐cell activation (typically via anti‐CD3/CD28 stimulation), gene transfer using retroviral or lentiviral vectors encoding the CAR construct, cellular expansion, and reinfusion following lymphodepleting chemotherapy, typically fludarabine plus cyclophosphamide. Lymphodepletion depletes regulatory and suppressive lymphocyte populations while inducing a cytokine milieu enriched in interleukin (IL)‐7 and IL‐15, thereby promoting homeostatic proliferation and persistence of the infused CAR T cells.

Upon antigen engagement, the CAR establishes a synthetic immune synapse that triggers ZAP70/SYK‐proximal signaling, leading to the activation of NF‐κB, NFAT, and AP‐1 pathways. Effector mechanisms include (i) perforin/granzyme‐mediated cytolysis and Fas–FasL interactions, (ii) secretion of pro‐inflammatory and T‐cell growth cytokines (e.g., IFN‐γ, TNF, IL‐2), and (iii) clonal expansion with the acquisition of memory characteristics. The co‐stimulatory domain critically shapes T‐cell metabolism and kinetics: CD28 costimulation favors rapid expansion, glycolytic metabolism, and effector differentiation, whereas 4‐1BB signaling enhances mitochondrial biogenesis, fatty‐acid oxidation, and central‐memory persistence. Treatment efficacy is further influenced by product attributes (naïve/central‐memory composition, transduction efficiency), host factors (age, fitness of the T‐cell repertoire), and tumor features (antigen density, microenvironmental resistance) [7].

**FIGURE 1:**
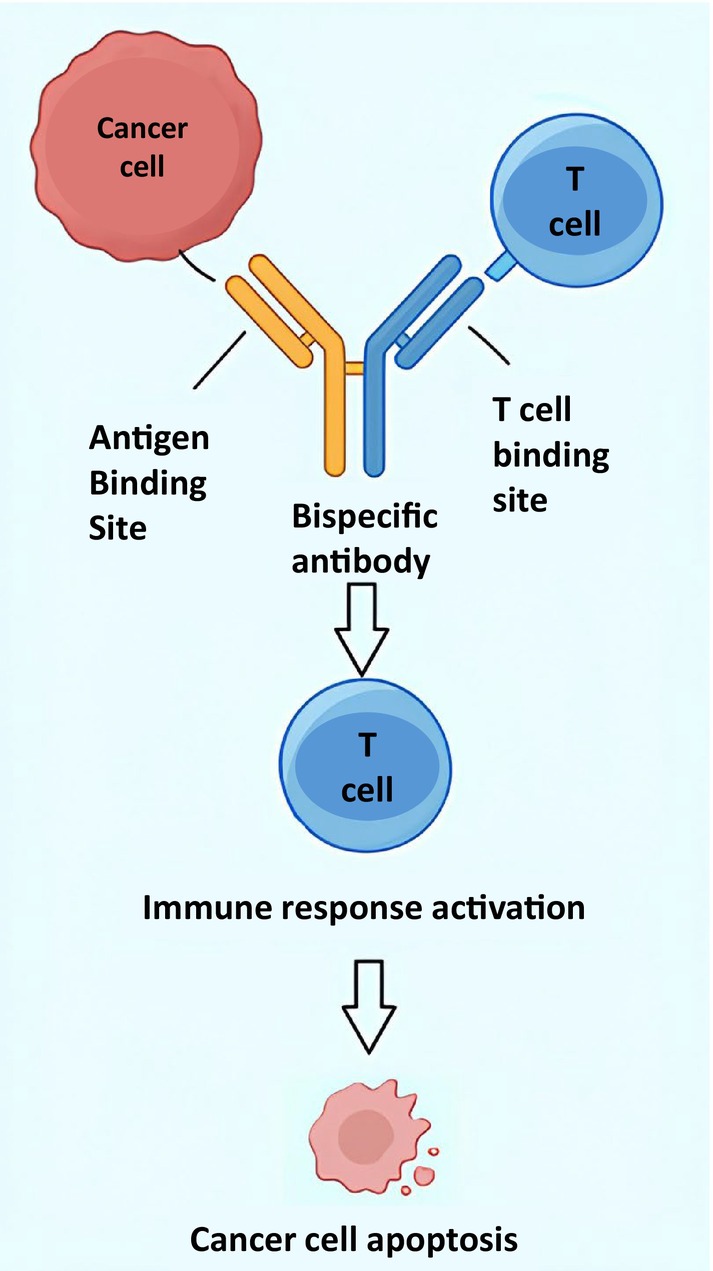
Mechanism of action of CAR‐T cell therapy.

MCL is a particularly compelling indication for CD19 CAR T cells. CD19 expression is preserved across classical and variant histologies; the disease is frequently systemic with marrow and blood involvement, enabling interaction with circulating effectors; and resistance to chemoimmunotherapy and BTK inhibition is common. In this setting, anti‐CD19 CAR T cells have achieved high response rates in patients refractory to BTK inhibition. Potential contributors to efficacy in MCL include disruption of BCR‐dependent survival signaling through direct cytolysis, robust CAR T‐cell expansion within lymphoid and blood compartments where MCL typically resides, and possible immunomodulatory effects of prior BTK inhibition, which may enhance CAR T‐cell function in some patients. Conversely, mechanisms of relapse include T‐cell exhaustion mediated by PD‐1/LAG‐3/TIM‐3 upregulation and metabolic stress, immunosuppressive influences of the tumor microenvironment, and modulation or loss of CD19 expression [8] (Figure 1).

### 2.2 Adverse Events

#### 2.2.1 Cytokine Release Syndrome (CRS)

CRS is the most common acute toxicity of CAR T‐cell therapy. Pathophysiologically, CAR T‐cell recognition of tumor cells induces a burst of IFN‐γ and other effector cytokines that activate monocytes/macrophages, which then amplify the inflammatory cascade through secretion of IL‐6, IL‐1, IL‐10, and TNF. Endothelial activation reflected by increased angiopoietin‐2 and von Willebrand factor, together with nitric oxide‐mediated vasodilation, drives capillary leak, hypotension, hypoxia, coagulopathy, and, in severe cases, a hemophagocytic lymphohistiocytosis (carHLH)‐like condition characterized by hyperferritinemia and hypofibrinogenemia. Clinically, CRS usually develops within the first few days after infusion—earlier with CD28‐based products, and somewhat later with 4‐1BB‐based products—and may range from isolated fever to life‐threatening shock and hypoxemic respiratory failure [9].

Risk factors include high tumor burden, rapid in vivo CAR T‐cell expansion, elevated baseline inflammatory markers, and possibly the use of CD28 costimulation. Grading is standardized according to ASTCT criteria, which define severity based on fever, hypotension/vasopressor requirement, and hypoxia. Management is stepwise: Grade 1 is treated with supportive (antipyretics, fluids, close monitoring), whereas Grade ≥ 2 requires anti‐IL‐6 receptor blockade (tocilizumab), with escalation to corticosteroids (e.g., dexamethasone) for persistent or worsening toxicity [10]. Refractory cases and HLH‐like CRS may benefit from IL‐1 blockade (anakinra) and intensive care support [11]. Concomitant infections must always be excluded and promptly treated, while high‐risk patients—such as those with rapid fever onset, rising ferritin or CRP, or brisk CAR T‐cell expansion—should be identified early for preemptive intervention (Table 1).

**TABLE 1:** Cytokine release syndrome (CRS) after CAR T‐cell therapy: pathophysiology, risk factors, clinical features, grading and management [10, 11].

CRS	
Pathophysiology	Tumor recognition by CAR T cells induces IFN‐γ and other cytokines, activating monocytes/macrophages → this amplifies inflammation through IL‐6, IL‐1, IL‐10, and TNF. Endothelial activation with angiopoietin‐2, von Willebrand factor, and nitric oxide release causes vasodilation, capillary leak, hypotension, hypoxia, and coagulopathy.
Timing	Within days of infusion; earlier with CD28‐based constructs, later with 4‐1BB‐based products.
Risk factors	High tumor burden; rapid CAR T‐cell expansion; elevated baseline CRP/ferritin; CD28 costimulation; high baseline inflammatory state.
Clinical spectrum	Fever, malaise, anorexia; hypotension, hypoxia, tachycardia; transaminitis, coagulopathy; severe cases with shock; and multiorgan dysfunction.
Grading	Ranges from Grade 1 (isolated fever) to Grade ≥ 4 (life‐threatening shock/organ failure).
Management	Grade 1: supportive care (antipyretics, IV fluids, oxygen). Grade ≥ 2: tocilizumab (anti‐IL‐6R) with escalation to corticosteroids if refractory. Anakinra (IL‐1 blockade) for refractory or HLH‐like cases; ICU care for hemodynamic/respiratory support; exclude and treat infections rigorously.
Monitoring and prevention	Close monitoring during the first 7–10 days postinfusion; identify high‐risk patients (rapid fever onset, CRP, brisk CAR expansion).
Differential diagnosis	Infection (bacterial/viral/fungal), sepsis, tumor lysis syndrome, allergic reactions, and engraftment syndrome.

#### 2.2.2 Immune Effector Cell‐Associated Neurotoxicity Syndrome (ICANS)

ICANS is a distinct, though sometimes overlapping, toxicity characterized by encephalopathy, attention and language deficits (e.g., expressive aphasia), tremor, seizures, and, rarely, cerebral edema. The predominant pathophysiology appears to involve diffuse endothelial activation with blood–brain barrier disruption, allowing cytokines and possibly immune cells to access the CNS. IL‐1 and GM‐CSF have been implicated, whereas IL‐6 seems to play a less central role within the CNS compartment. This may explain why tocilizumab—effective for CRS—does not reliably control isolated ICANS and, in some cases, may exacerbate symptoms by enhancing unbound IL‐6 trafficking. Risk factors include high tumor burden, severe or early onset CRS, CD28‐based co‐stimulation, older age, and preexisting neurological disease comorbidities [12].

Assessment relies on the immune effector cell encephalopathy (ICE) score, which evaluates orientation, naming, command following, writing, and attention. ASTCT grading incorporates additional parameters, including level of consciousness, seizures, motor findings, and evidence of intracranial pressure or edema. Management is centered on corticosteroids (e.g., dexamethasone or methylprednisolone) as first‐line therapy, with levetiracetam recommended for seizure prophylaxis [13]. For steroid‐refractory neurotoxicity cases, anakinra is increasingly employed, aggressive ICU‐level care while intensive neurocritical care—including airway protection and intracranial pressure management with hyperosmolar therapy—may be required in severe presentations. Tocilizumab should be reserved for patients with concurrent CRS rather than those with isolated ICANS [14] (Table 2).

**TABLE 2:** Immune effector cell‐associated neurotoxicity syndrome (ICANS) after CAR T‐cell therapy: pathophysiology, risk factors, clinical features, grading, and management [12, 13, 14].

ICANS	
Pathophysiology	Endothelial activation and blood–brain barrier disruption permit cytokines and immune cells to enter the CNS.
Timing	Within the first week after CAR T‐cell infusion, often concurrent with or following CRS, but may also arise independently.
Risk factors	High tumor burden; early/severe CRS; CD28‐based constructs; older age; preexisting neurological disease or CNS involvement.
Clinical spectrum	Encephalopathy, attention and language deficits, tremor, dysgraphia, impaired handwriting, seizures, somnolence, and rarely, fatal cerebral edema.
Grading	ICE score (orientation, naming, commands, writing, attention); ASTCT criteria include level of consciousness, seizures, motor findings, raised intracranial pressure or cerebral edema.
Management	Corticosteroids are the mainstay; levetiracetam is commonly used for seizure prophylaxis. Anakinra for steroid‐refractory cases; intensive neurocritical care with airway protection, ICP management (e.g., hyperosmolar therapy). Tocilizumab is reserved only if concurrent CRS is present.
Monitoring and prevention	Frequent neurological assessments during the first 10–14 days; preemptive seizure prophylaxis; identify patients at high risk (older age, early CRS, neurologic comorbidities).
Differential diagnosis	CNS infection (viral, fungal, bacterial), metabolic encephalopathy, stroke, drug‐related neurotoxicity, and leptomeningeal disease.

#### 2.2.3 Others

Prolonged cytopenias, lasting weeks to months, are common after CAR T‐cell therapy and may result from marrow injury, systemic inflammation, or the cumulative effects of prior therapies. These cytopenias increase the risk of infections and often necessitate growth factor support and transfusions. B‐cell aplasia and hypogammaglobulinemia represent expected on‐target effects, predisposing patients to bacterial and viral infections. Standard supportive measures include intravenous immunoglobulin replacement and antimicrobial prophylaxis, while reactivation of latent viruses (e.g., HBV) should be prevented according to established guidelines [15].

### 2.3 Brexucabtagene Autoleucel

Brexucabtagene autoleucel (brexu‐cel, KTE‐X19) is the first CAR T‐cell product approved for R/R MCL, representing a major advance in the therapeutic landscape. Its approval was based on the pivotal phase II ZUMA‐2 trial, which enrolled heavily pretreated patients who had previously received anthracycline‐ or bendamustine‐based chemotherapy, an anti‐CD20 antibody, and a BTKi [16]. In the study, patients underwent leukapheresis, optional bridging therapy, lymphodepleting chemotherapy with fludarabine and cyclophosphamide, followed by a single infusion of brexu‐cel at 2 × 10^6^ CAR T cells/kg.

In the primary efficacy analysis, brexu‐cel achieved an overall response rate (ORR) of 93% with a CR rate of 67%. Responses were consistent across high‐risk subgroups, including patients with BTKi‐refractory disease, TP53 aberrations, blastoid morphology, and high proliferative index (Ki‐67 ≥ 30%). With a median follow‐up of 3 years, median progression‐free survival (PFS) was 25.8 months and median overall survival (OS) was 46.6 months, with approximately one‐quarter of patients maintaining long‐term remission. However, no survival plateau has yet been observed, indicating a persistent risk of relapse despite deep initial responses [17].

*Toxicity evaluation revealed* a high incidence of treatment‐related adverse events. Nearly all patients (99%) experienced Grade ≥ 3 toxicities, most commonly prolonged cytopenias (> 90 days in some cases). Severe infections were also frequent, with ≥ Grade 3 infections in 32%–44% of patients, including fatal events, highlighting the need for long‐term antimicrobial prophylaxis and immunoglobulin replacement [18].

CRS occurred in 91% of patients, typically within 2 days postinfusion, presenting with fever and hypotension to hypoxia, sometimes requiring intensive care support. Tocilizumab was administered in 59% of cases, while corticosteroids were reserved for higher‐grade events.

ICANS was reported in 63% of patients, with Grade ≥ 3 in approximately one‐third, including rare but severe cases of cerebral edema requiring neurosurgical intervention. Clinical features included encephalopathy, language dysfunction, and seizures, managed with corticosteroids and neurocritical supportive care.

Real‐world data from the US CAR‐T Consortium and the Center for International Blood and Marrow Transplant Research (CIBMTR) registries showed that many patients would not have met ZUMA‐2 eligibility due to comorbidities, disease burden, or prior therapies. At 12 months, PFS ranged from 59% to 61%, and OS exceeded 80% in several series. The toxicity profile was consistent with ZUMA‐2, with CRS reported in 85%–90% and ICANS in 30%–32% of patients. Non‐relapse mortality at 1 year was ~9%, primarily infection‐related [19].

Overall, brexu‐cel has demonstrated unprecedented activity in R/R MCL, including in patients with high‐risk biological features such as TP53 mutation, blastoid histology, or BTKi refractoriness.

Further information was provided by the ZUMA‐18 expanded access protocol (NCT04162756), which included patients ineligible for prior trials or awaiting commercial availability. The study also evaluated products manufactured with parameters marginally outside Food and Drug Administration (FDA)‐defined release specifications (e.g., cell viability < 80%). Twenty‐three patients were treated. Compared with ZUMA‐2, the ZUMA‐18 cohort was older (median age 69 vs. 64–65 years), more heavily pretreated (median 4 prior lines), and had greater baseline functional impairment (ECOG PS 1 in 60% vs. 35% in ZUMA‐2), reflecting a frailer, higher‐risk population.

Despite these adverse characteristics, brexu‐cel maintained meaningful clinical activity, with a CR rate of 57% and a slightly shorter median duration of response compared to ZUMA‐2. Notably, the 3‐year OS rate reached 58%, underscoring the durable efficacy of CAR T‐cell therapy even in this vulnerable population.

ZUMA‐18 reinforces the pivotal ZUMA‐2 findings, demonstrating consistent efficacy and manageable safety in a less selected, real‐world MCL population. These data further establish brexu‐cel as a viable treatment option for patients with R/R MCL, including those with advanced age, comorbidities, and extensive prior therapy [20].

### 2.4 Lisocabtagene Maraleucel

Lisocabtagene maraleucel (liso‐cel) is an autologous CD19‐directed CAR‐T cell therapy that incorporates a defined CD4/CD8 composition and a 4‐1BB co‐stimulatory domain. Recently approved for R/R MCL, it was evaluated in the pivotal TRANSCEND NHL 001 trial, which demonstrated robust clinical activity with a comparatively favorable safety profile, particularly regarding severe CRS and ICANS [21, 22].

In the MCL cohort of TRANSCEND, 92 patients received liso‐cel infusion following leukapheresis, with a median follow‐up of 16.1 months. The study population was enriched for high‐risk disease: 75% had a Ki‐67 ≥ 30%, 31% displayed blastoid morphology, and 23% carried TP53 mutations. Nearly all patients (94%) had received prior BTK inhibitor therapy. Despite these adverse‐risk features, the ORR was 83.1%, with a durable CR rate of 72.3%. Encouragingly, meaningful activity was also observed in patients with secondary central nervous system (CNS) involvement, with an ORR of 86% and a CR rate of 71% [23].

From a safety standpoint, liso‐cel demonstrated lower toxicity compared to brexu‐cel. The incidence of CRS was 61%, but Grade ≥ 3 events occurred in only 1%. ICANS was observed in 31% of patients, with Grade ≥ 3 in 9%. Importantly, no Grade‐5 CRS/ICANS events were reported. Severe infections (Grade ≥ 3) were documented in 15% of cases, consistent with CAR T‐related immunosuppression but lower than historical benchmarks with brexu‐cel. Collectively, these findings established liso‐cel as a highly effective and relatively safer CAR‐T option in R/R MCL, particularly in patients exposed to prior BTK inhibitors, although outcomes in this subgroup remain suboptimal and highlight an ongoing need for therapeutic optimization.

Nevertheless, resistance remains a clinically relevant challenge. Relapse may arise from unfavorable tumor microenvironmental conditions, loss of target antigen expression (e.g., CD19 downregulation), or adverse genomic alterations such as TP53 mutations. Ongoing research is exploring strategies to overcome these mechanisms, including dual‐antigen targeting (e.g., CD19/CD22 or CD19/BAFF‐R), trispecific CAR constructs, and rational combinatorial approaches integrating CAR T‐cell therapy with small‐molecule inhibitors or bispecific antibodies (BsAbs) [24].

Future studies will be critical to define the optimal sequencing of CAR‐T with BTK inhibitors and novel BsAbs, and to determine whether earlier integration of these modalities in the disease course can further improve long‐term outcomes.

### 2.5 Ongoing Clinical Trials

#### 2.5.1 Relmacabtagene Autoleucel

Relmacabtagene autoleucel (relma‐cel) is an autologous, CD19‐directed CAR T‐cell therapy currently under clinical evaluation for R/R MCL in the phase II NCT04718883 trial. Structurally, relma‐cel incorporates a second‐generation CAR backbone with a CD3ζ activation domain and a 4‐1BB costimulatory signal, designed to optimize T‐cell expansion, persistence, and memory formation while mitigating excessive early cytokine release. Mechanistically, this construct more closely resembles lisocabtagene maraleucel than brexucabtagene autoleucel, the latter relying on CD28 costimulation, which drives more rapid but often less durable T‐cell proliferation.

Preliminary efficacy data suggest that relma‐cel induces meaningful responses in heavily pretreated patients, including those previously exposed to BTKi—a population typically characterized by chemoresistant disease and poor prognosis. Early reports indicate overall and complete response rates comparable to other CD19‐directed CAR T‐cell therapies, with encouraging evidence of durability; however, extended follow‐up is required to confirm sustained benefit [25].

In terms of safety, the incidence of CRS and ICANS appears manageable and consistent with other 4‐1BB‐based constructs. CRS has generally been low‐grade and responsive to standard interventions such as tocilizumab and supportive care, while ICANS events have been less frequent, typically reversible with corticosteroid therapy. The defined CAR T‐cell composition and controlled manufacturing process may contribute to the favorable safety profile observed thus far.

Overall, relma‐cel represents a promising investigational option for patients with R/R MCL, potentially offering a favorable balance between durable efficacy and reduced acute toxicity. Pending regulatory review, its ultimate role in clinical practice will depend on the maturity of efficacy data, comparative outcomes versus approved CAR T‐cell products such as brexu‐cel and liso‐cel, and integration with emerging therapeutic modalities including BsAbs and antibody–drug conjugates.

#### 2.5.2 Combination Strategies

The integration of brexu‐cel with targeted agents is an area of growing interest, aimed at optimizing CAR T‐cell efficacy and durability. One such strategy involves combination with the BTKi ibrutinib, currently being evaluated in the phase II clinical study CARMAN (NCT06482684) [26]. Beyond its established role in BCR signaling, ibrutinib exerts immunomodulatory effects, including enhancement of T‐cell function, reduction of exhaustion phenotypes, and improvement in metabolic fitness.

Preclinical models indicate that ibrutinib may create a more favorable immune microenvironment by attenuating inhibitory signaling pathways and supporting T‐cell expansion, potentially enhancing CAR T cells' persistence and antitumor activity. In this context, a phase II clinical trial is comparing first‐line treatment with KTE‐X19 following a shortened induction with rituximab and ibrutinib against conventional immunochemotherapy plus ibrutinib followed by ASCT in younger patients with high‐risk MCL. For elderly but otherwise fit patients who remain eligible for CAR T‐cell therapy, the comparator arm consists of immunochemotherapy plus a BTKi. Patients who fail to achieve at least a partial response after induction may receive two additional cycles of R‐CHOP, though these cycles can be omitted in cases where adequate response is achieved with ibrutinib‐based therapy. The results of this trial are expected to provide insights into the feasibility of early CAR T‐cell intervention, the potential to overcome limitations of standard therapy, and the synergistic role of BTK inhibition in enhancing T‐cell fitness and persistence.

Another approach involves sequential administration of acalabrutinib, a selective second‐generation BTK inhibitor, in combination with rituximab, followed by brexu‐cel infusion. The rationale for this strategy lies in the ability of acalabrutinib and rituximab to provide effective cytoreduction while preserving a favorable immune microenvironment, which may improve CAR T‐cell manufacturing feasibility as well as in vivo expansion and persistence. The strategy is under investigation in an *early phase I* clinical trial (NCT05495464) in patients with previously untreated high‐risk MCL.

The trial primarily evaluates the safety and tolerability of acalabrutinib plus rituximab induction, followed by brexu‐cel infusion. Secondary endpoints include efficacy measures such as CR rate and PFS, duration of response, and CAR T‐cell kinetics. These data will clarify whether early integration of targeted therapy with CAR T‐cells can induce deeper and more durable remissions compared with traditional frontline approaches.

A further construct, CD19R(EQ)28zetaEGFRt + Tn/Tmem, enriched in naïve and memory T‐cell subsets, is currently under clinical investigation in combination with acalabrutinib (NCT04484012). Less differentiated T‐cell populations exhibit superior proliferative capacity, metabolic fitness, and long‐term persistence, key determinants of sustained CAR‐T functionality and durable remission. Preclinical evidence shows that CAR‐T products enriched in naïve and memory‐like cells display enhanced expansion kinetics and resistance to exhaustion, potentially translating into more robust clinical outcomes. Acalabrutinib, beyond its established role in targeting BCR signaling, may provide additional synergistic effects by modulating the tumor microenvironment, reducing immunosuppressive signaling, and promoting T‐cell activity, thereby optimizing cytotoxicity and minimizing relapse risk.

Finally, ONCT‐808, a next‐generation autologous CAR T‐cell therapy, is designed to achieve enhanced expansion, persistence, and functional durability relative to earlier CAR‐T constructs. Its development focuses on improving T‐cell quality, engraftment, longevity, and resistance to functional exhaustion in the tumor microenvironment. ONCT‐808 incorporates an optimized cellular composition and manufacturing process to enrich for less differentiated T‐cell subsets with superior proliferative capacity and long‐term memory potential [18, 27].

Table 3 provides a comprehensive overview of pivotal and ongoing clinical trials evaluating CAR‐T therapies in MCL, highlighting both their efficacy—characterized by high response rates and durable remissions—and their safety profile, even in patients with high‐risk features or BTKi‐refractory disease (Table 3).

**TABLE 3:** Clinical trials of CAR‐T cell therapy in relapsed/refractory mantle cell lymphoma.

CAR‐T product	Trial/phase	Setting	Key efficacy outcomes	Safety profile
Brexu‐cel	ZUMA‐2 (Phase II)	Heavily pretreated	ORR 93%, CR 67%; median PFS 25.8 months; OS 46.6 months; 3‐year follow‐up: ~25% in long‐term remission	CRS 91% (G ≥ 3: 15%); ICANS 63% (G ≥ 3: 31%); prolonged cytopenias > 90 days; G ≥ 3 infections 32%–44%
Brexu‐cel	ZUMA‐18 (EAP)	Frailer pts.	CR 57%; shorter median DoR vs. ZUMA‐2; 3‐year OS 58%	Toxicity consistent with ZUMA‐2; manageable
Liso‐cel	TRANSCEND‐NHL‐001 (Phase I/II, MCL cohort)	High‐risk features	ORR 83.1%, CR 72.3%; durable responses; CNS involvement: ORR 86%, CR 71%	CRS 61% (G ≥ 3: 1%); ICANS 31% (G ≥ 3: 9%); G ≥ 3 infections 15%; no Grade 5 CRS/ICANS
Rlma‐cel	NCT04718883 (Phase II, ongoing)	Prior BTKi	Preliminary ORR/CR rates encouraging; durability under follow‐up	CRS mostly low grade, responsive; ICANS infrequent, reversible
Brexu‐cel + ibrutinib	CARMAN trial (NCT06482684, Phase II, ongoing)	High‐risk untreated or R/R	Efficacy endpoints pending	Ibrutinib may enhance T‐cell persistence, reduce exhaustion
Brexu‐cel + acalabrutinib + rituximab	NCT05495464 (Phase I, ongoing)	High‐risk, untreated	Primary: safety/tolerability; secondary: CR, PFS, CAR‐T kinetics	Early data pending
CD19CAR‐CD28‐CD3zeta‐Egfrt‐expressing Tn/mem‐enriched T‐lymphocytes + acalabrutinib	NCT04484012 (Phase I, ongoing)	R/R MCL	Data pending	Designed to improve persistence via naïve/memory enrichment
ONCT‐808	Early clinical development	MCL cohorts planned	Aimed to enhance expansion, persistence, and durability	Expected improved safety and resistance to exhaustion

## 3 BsAbs

### 3.1 Mechanism of Action

BsAbs have emerged as a transformative immunotherapeutic strategy in MCL, leveraging the patient's own immune system to selectively target malignant B cells. These molecules are engineered to simultaneously recognize two antigens: CD20 on lymphoma cells and CD3 on T lymphocytes. By bridging T cells and tumor cells, BsAbs form a synthetic immunological synapse that triggers robust T‐cell activation and proliferation. Activated T cells release cytotoxic granules containing perforin and granzymes, inducing apoptosis in the CD20‐positive target cells. In addition, BsAb engagement promotes the secretion of proinflammatory cytokines such as interferon‐γ, tumor necrosis factor‐α, and IL‐2, which further recruit and amplify effector T‐cell responses within the tumor microenvironment [16].

**FIGURE 2:**
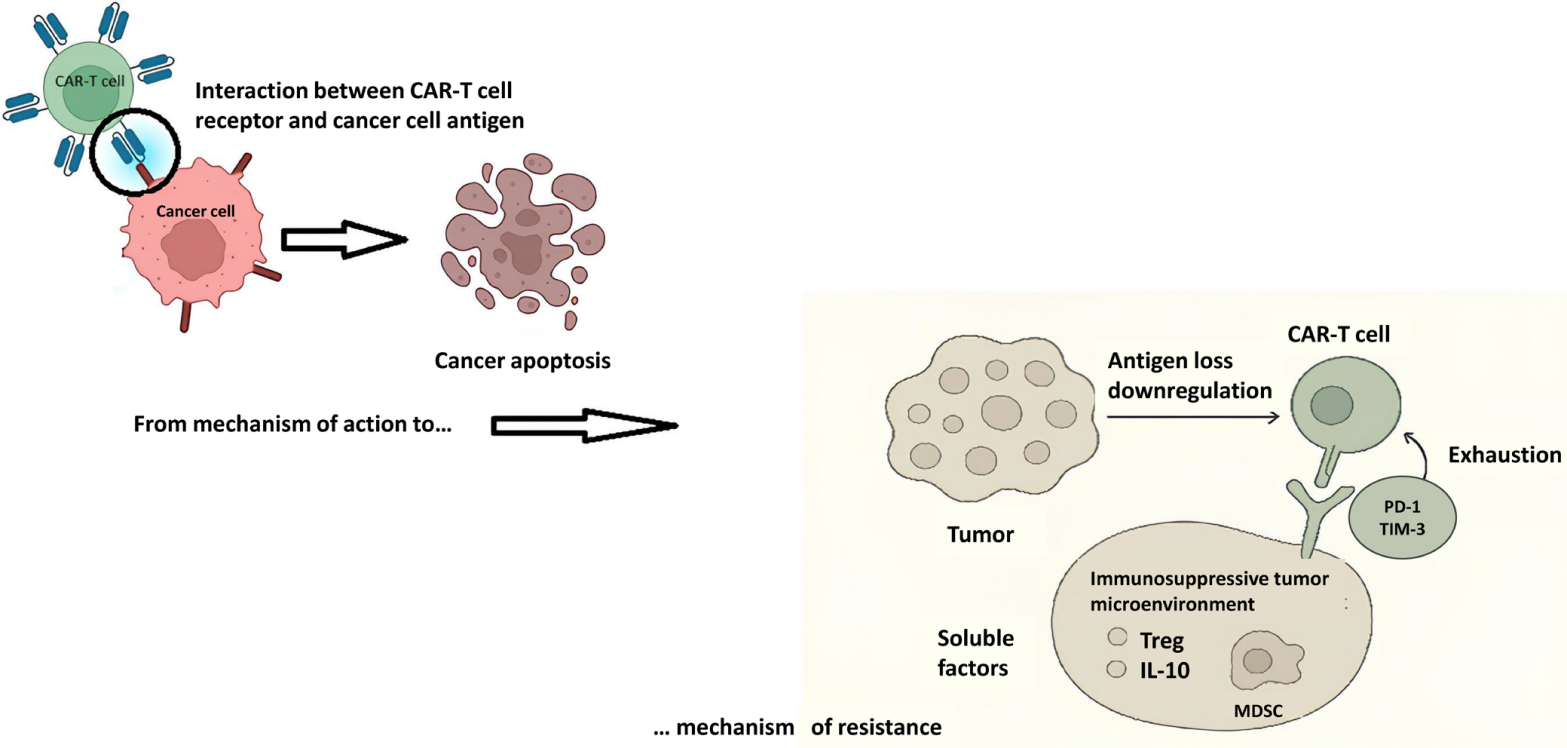
Bispecific antibodies and mechanisms of resistance. Tumor cells may evade CAR‐T recognition through antigen loss or downregulation, reducing target availability on the cell surface. CAR‐T cell exhaustion, characterized by the upregulation of inhibitory receptors such as PD‐1 and TIM‐3, impairs effector function and persistence. Additionally, the immunosuppressive tumor microenvironment—comprising regulatory T cells (Tregs), myeloid‐derived suppressor cells (MDSCs), and soluble factors such as IL‐10—further suppresses CAR‐T cell activity and limits therapeutic efficacy.

Unlike conventional monoclonal antibodies, BsAbs act independently of major histocompatibility complex‐mediated antigen presentation, enabling direct and rapid tumor cell lysis. Their capacity for serial killing allows a single T cell to eliminate multiple target cells, potentially enhancing both the depth and durability of the response. However, this potent immune activation also underlies the characteristic toxicities of BsAbs, primarily CRS and, less commonly, ICANS. To mitigate these risks, clinical protocols often employ step‐up dosing, corticosteroids premedication, and pretreatment with anti‐CD20 antibodies to reduce tumor burden prior to BsAb administration [14]. Overall, the mechanistic design of BsAbs provides a highly targeted, “off‐the‐shelf” approach to engage cytotoxic T cells against MCL, offering the potential for rapid and potent antitumor activity even in patients with relapsed or refractory disease (Figure 2).

### 3.2 Adverse Events

#### 3.2.1 CRS and ICANS

CRS is the most frequently observed toxicity of BsAbs, resulting from rapid and extensive T‐cell activation upon simultaneous engagement of CD3 on T lymphocytes and CD20 on malignant B cells. In clinical studies of glofitamab and mosunetuzumab in MCL, CRS has been reported in 50%–75% of patients. Most events are Grade 1–2 and manageable with supportive care; however, higher‐grade CRS (Grade ≥ 3) can occur, particularly in patients with high tumor burden or during rapid step‐up dosing. Severe CRS may require hospitalization and prompt intervention. Management strategies include IL‐6 receptor antagonists (e.g., tocilizumab), corticosteroids, temporary interruption of BsAb therapy, and close monitoring of vital signs and laboratory parameters [10].

ICANS represents another clinically significant adverse effect, though less common than CRS. In reported studies of BsAb therapy in MCL, ICANS events were generally Grades 1–2, transient, and reversible with supportive care. Nevertheless, careful neurological monitoring is essential during therapy to detect and manage neurotoxicity promptly [12].

#### 3.2.2 Tumor Lysis Syndrome (TLS)

TLS is another potential complication of BsAb therapy, particularly in patients with a high tumor burden or rapidly proliferating disease. TLS results from abrupt and extensive tumor cell destruction, leading to the release of intracellular electrolytes and nucleic acids.

Clinically, it manifests as hyperuricemia, hyperkalemia, hyperphosphatemia, and hypocalcemia, which can precipitate acute kidney injury, cardiac arrhythmias, seizures, and, in severe cases, multi‐organ dysfunction. The reported incidence among heavily pretreated patients receiving CD3‐engaging BsAbs ranges from 0% to 11% [28].

Prophylactic measures to mitigate TLS risk include aggressive hydration, frequent monitoring of serum electrolytes and renal function, and administration of urate‐lowering agents such as allopurinol or rasburicase. Additionally, step‐up dosing of BsAbs and pretreatment with anti‐CD20 antibodies can limit the initial tumor cell kill, thereby attenuating the severity of TLS.

#### 3.2.3 Others

Additional toxicities associated with BsAb therapy in MCL encompass hematologic, infectious, metabolic, and infusion‐related events. Hematologic adverse events are particularly common and include neutropenia, anemia, and thrombocytopenia. These cytopenias are often more pronounced in heavily pretreated patients due to cumulative marrow suppression from prior chemotherapies, targeted agents, or ASCT. Neutropenia, in particular, increases the risk for opportunistic infections, while anemia and thrombocytopenia can contribute to fatigue, bleeding, and reduced quality of life. Management strategies typically involve dose adjustments, growth factor support (e.g., granulocyte‐colony stimulating factor), transfusion support for red blood cells or platelets, and close laboratory monitoring to guide intervention [14].

Infections remain a critical concern in BsAb‐treated MCL patients, arising both from transient immunosuppression and from prior therapy‐related lymphodepletion or BsAb‐mediated cytotoxicity. Bacterial, viral, and, less commonly, fungal infections have all been reported, ranging from mild upper respiratory tract infections to life‐threatening sepsis. Early recognition, prompt initiation of appropriate antimicrobial therapy, and, in selected cases, prophylactic antimicrobials or antivirals are essential to reduce morbidity and prevent treatment interruptions. Vaccination status should be reviewed prior to therapy initiation, and patients should receive counseling on infection prevention strategies [29].

Infusion‐related reactions (IRRs) are another common toxicity, particularly, during initial BsAb infusions. IRRs typically manifest as fever, chills, rigors, hypotension, rash, or mild dyspnea. Most reactions are Grades 1–2 and self‐limiting, but severe cases can occur, especially if infusion rates are not carefully titrated. Standard management includes premedication with antipyretics, antihistamines, and corticosteroids, temporary interruption or slowing of the infusion, and close monitoring of vital signs. Staff education and preparedness are essential to recognize and respond promptly to IRRs, thereby minimizing risk and maintaining treatment continuity.

### 3.3 Ongoing Clinical Trials

#### 3.3.1 Glofitamab

Glofitamab is a CD20 × CD3 BsAb designed with a unique 2:1 configuration that enables bivalent binding to CD20 on B cells and monovalent engagement of CD3 on T cells. This design facilitates potent T‐cell activation and targeted cytotoxicity against malignant B cells. Initially approved for patients with R/R DLBCL after multiple prior therapies, glofitamab's activity has also been investigated in MCL. In the phase I/II NP30179 trial (NCT03075696), 61 heavily pretreated MCL patients received step‐up glofitamab dosing following obinutuzumab pretreatment to mitigate CRS. Obinutuzumab was administered either as a single 1 g infusion or a split 2 g dose, with the latter regimen associated with lower CRS incidence and severity. Glofitamab dosing escalated during the first cycle (2.5 mg on Day 8; 10 mg on Day 15), reaching a fixed dose of 16–30 mg every 3 weeks for up to 12 cycles. Patients had received a median of two prior lines of therapy, with over half previously exposed to BTK inhibitors and more than one‐third to bendamustine [18].

With a median follow‐up of 19.6 months, glofitamab demonstrated robust efficacy in this high‐risk population, with an ORR of 85% and a CR rate of 78%. Responses were rapid and durable, with a PFS and OS of 16.8 and 29.9 months, respectively, and better outcomes were observed in BTKi‐naïve patients, whereas prior bendamustine exposure was associated with somewhat attenuated responses. The safety profile was manageable. The most frequent adverse events included CRS (70%), neutropenia (38%), COVID‐19 infections (32%), and pyrexia (32%). ICANS was uncommon, exclusively low grade, and fully reversible. Treatment discontinuation occurred in 60% of patients, largely due to cytopenias, CRS, or infections. Eight deaths were reported, six of which were attributable to COVID‐19 during the pandemic period.

Overall, fixed‐duration glofitamab monotherapy demonstrated substantial activity with a manageable safety profile in heavily pretreated MCL, including BTKi‐exposed patients. The ongoing phase III GLOBRYTE study will further define its role by comparing glofitamab with bendamustine–rituximab or lenalidomide–rituximab in patients with R/R MCL previously treated with BTK inhibitors [30].

#### 3.3.2 Mosunetuzumab

Mosunetuzumab is a humanized IgG1 BsAb that simultaneously targets CD20 on B cells and CD3 on T cells. Its Fc domain is engineered to prevent interaction with Fcγ receptors and complement, while the construct contains a single binding epitope for CD20—similar to rituximab—and one for CD3, enabling selective T‐cell redirection toward malignant B cells. Administration follows a step‐up dosing schedule to minimize the risk and severity of CRS. Initially developed in R/R FL and other indolent NHLs, where it showed high efficacy, mosunetuzumab has also been explored in MCL, particularly in the challenging population refractory to BTK inhibitors [18, 31, 32]. Early clinical data suggest that mosunetuzumab can elicit meaningful responses in this high‐risk population while maintaining a manageable safety profile, supporting its ongoing investigation in larger studies.

#### 3.3.3 Epcoritamab

Epcoritamab is a full‐length IgG1 BsAb administered subcutaneously, designed to redirect CD3‐positive T cells toward CD20‐expressing malignant B cells. Clinical studies have demonstrated substantial efficacy both as monotherapy and in combination regimens for R/R DLBCL and FL, leading to regulatory approval in several countries for patients with R/R DLBCL after at least two prior lines of therapy.

Data regarding its activity in MCL, however, remains limited. In the dose‐escalation phase of a first‐in‐human study enrolling 73 patients with R/R NHLs, only four MCL cases were included, precluding definitive conclusions on its clinical utility in this disease [18, 33, 34].

Given the aggressive biology of BTK inhibitor–refractory MCL and the encouraging efficacy of epcoritamab in other B‐cell malignancies, dedicated clinical trials are warranted to clarify its therapeutic potential and safety profile in this patient population.

#### 3.3.4 Odronextamab

Odronextamab is a hinge‐stabilized, fully human IgG4‐based BsAb that targets CD20 on malignant B cells and CD3 on T cells, thereby promoting T‐cell–mediated cytotoxicity [35]. Clinical studies have demonstrated significant activity of odronextamab in R/R DLBCL and FL, including responses in patients previously exposed to both anti‐CD20 antibodies and CAR‐T therapy [18, 36, 37]. While these promising results have led to advanced‐phase trials in both indolent and aggressive B‐cell lymphomas, data on MCL remain limited. Early‐phase studies of R/R NHL included small numbers of MCL cases, suggesting potential activity but insufficient data.

#### 3.3.5 Combination Strategies

Given the suboptimal durability of responses observed with BsAbs as monotherapy in R/R MCL, current research is focused on combinatorial approaches designed to potentiate antitumor activity and overcome resistance. Several ongoing trials are exploring glofitamab in multi‐agent regimens. The phase II GLOASIS trial (NCT06558604) is investigating the triplet of glofitamab, zanubrutinib, and venetoclax, integrating BTK inhibition, BCL2 blockade, and T‐cell redirection to achieve deeper and more durable remissions. Other studies are testing glofitamab with novel BTK inhibitors, such as pirtobrutinib (NCT06252675), which retains activity in covalent BTKi‐resistant disease, or with the immunomodulatory agent lenalidomide (NCT06192888) to augment T‐cell activation and enhance BsAb efficacy. Additional triplet strategies under investigation include glofitamab with ibrutinib plus obinutuzumab (NCT06357676) or acalabrutinib plus obinutuzumab (NCT06054776), exploiting synergistic B‐cell depletion and immune modulation. A pivotal randomized phase III trial (NCT06084936) is comparing glofitamab monotherapy with rituximab‐bendamustine or rituximab‐lenalidomide, providing a head‐to‐head assessment against standard‐of‐care chemoimmunotherapy regimens.

Beyond glofitamab, other BsAbs are being evaluated in combination regimens for MCL. Mosunetuzumab is under investigation with polatuzumab vedotin (NCT06453044), leveraging T‐cell engagement alongside targeted cytotoxic delivery. This combination has shown promising results in R/R MCL, with an ORR of 88% and a CR rate of 79% in the study. Notably, efficacy was observed across high‐risk subgroups, including patients with prior CAR T‐cell therapy, TP53 mutations, and those with Ki‐67 scores ≥ 50%.

Based on these early‐phase findings, this combination has recently been incorporated into US treatment guidelines as a therapeutic option for R/R MCL, reflecting both its efficacy and manageable safety profile.

Epcoritamab is being explored in combination with ibrutinib (NCT07082868) or in a triplet with ibrutinib and venetoclax (NCT05283720), strategies designed to simultaneously disrupt BCR and BCL2 signaling while redirecting T‐cell cytotoxicity.

Collectively, these trials reflect a paradigm shift in R/R MCL management, positioning BsAbs not only as monotherapy but also as integral components of rationally designed combination regimens [18].

To better illustrate the efficacy and safety data of BsAbs in MC, Table 4 summarizes the main clinical results and treatment‐related toxicities reported across early‐phase studies (Table 4).

**TABLE 4:** Clinical outcomes and adverse events of bispecific antibodies in relapsed/refractory mantle cell lymphoma (MCL).

Agent	Population/trial	Efficacy (ORR/CR, survival)	Key adverse events	Notes
Glofitamab	61 pts., NP30179, BTKi‐exposed/naïve	ORR 85%, CR 78%, PFS 16.8 months, OS 29.9 months	CRS 70%, neutropenia 38%, infections (COVID‐19) 32%, ICANS rare	Better outcomes in BTKi‐naïve; reduced responses postbendamustine
Mosunetuzumab	Early‐phase, R/R NHL including BTKi‐refractory MCL	Encouraging efficacy in BTKi‐refractory MCL (limited data)	CRS (low grade), cytopenias, infections	Step‐up dosing is required to mitigate CRS
Epcoritamab	First‐in‐human dose escalation, 73 NHL (4 MCL)	Active in DLBCL/FL; only 4 MCL pts., no definitive conclusions	CRS (mostly Grades 1–2), cytopenias, infections	Subcutaneous; data in MCL limited
Odronextamab	Early‐phase NHL, including MCL	Responses in DLBCL/FL; limited MCL activity reported	CRS, infections, cytopenias	Hinge‐stabilized IgG4 BsAb

Abbreviations: BTKi, Bruton tyrosine kinase inhibitor; CR, complete response; CRS, cytokine release syndrome; DoR, duration of response; ICANS, immune effector cell‐associated neurotoxicity syndrome; NHL, non‐Hodgkin lymphoma; ORR, overall response rate; OS, overall survival; PFS, progression‐free survival.

## 4 Future Perspectives and Possible Therapeutic Sequencing

The rapidly evolving immunotherapy landscape in MCL underscores the critical need to define optimal sequencing strategies between different T‐cell redirecting approaches [38].

CAR‐T therapy has demonstrated high ORR and durable remissions even in heavily pretreated patients, positioning it as a potentially curative option for selected cases. However, CAR‐T therapy is associated with logistical and clinical challenges, including manufacturing time, the need for bridging therapy, and the risk of severe CRS and neurotoxicity, which may limit its applicability in frail or rapidly progressive patients [39, 40].

BsAbs provide a complementary immunotherapeutic approach, offering advantages such as outpatient administration, rapid initiation, repeat dosing, and generally manageable toxicity profiles.

Emerging evidence indicates that sequential use of CAR‐T and BsAbs is feasible. BsAbs can induce meaningful responses in patients relapsing after CAR‐T therapy, and conversely, CAR‐T can be effective following BsAb exposure, although prior CAR‐T may impact T‐cell fitness and the depth of subsequent response.

Preclinical studies have demonstrated that continuous stimulation of T cells with BsAbs over 28 days can induce T‐cell exhaustion, impairing antileukemic activity. Specifically, exposure to AMG 562, a CD19 × CD3 BsAb, led to a progressive decline in T‐cell function, with specific lysis decreasing from 88.4% on Day 7 to 8.6% on Day 28 (*p* = 0.0003). This exhaustion was associated with upregulation of inhibitory receptors, including PD‐1, Tim‐3, and LAG‐3, and downregulation of T‐cell receptor (TCR) expression. Importantly, introducing treatment‐free intervals (TFIs) by withdrawing AMG 562, restored T‐cell function, with specific lysis returning to 93.4% on day 14 (*p* < 0.0001). These findings underscore the importance of immunotherapy sequencing, as prior BsAb exposure may influence T‐cell fitness and the efficacy of subsequent treatments [41].

Future strategies may involve using CAR‐T as consolidation following a BsAb‐induced remission or employing BsAbs post‐CAR‐T relapse, with treatment decisions guided by disease kinetics, patient comorbidities, prior treatment history, and logistical considerations. In parallel, next‐generation immunotherapeutic constructs—including CARs with optimized costimulatory domains, dual‐antigen targeting, or “armored” designs capable of modulating the tumor microenvironment—have the potential to deepen responses, overcome immune escape, and broaden applicability in high‐risk MCL [42].

## 5 Conclusions

The therapeutic landscape of R/R MCL has been transformed by T‐cell‐redirecting immunotherapies, including both BsAbs and CAR‐T therapies [42].

CAR‐T therapies, such as brexu‐cel and liso‐cel, induce potent, deep, and durable remissions, even in patients with high‐risk features or BTKi‐refractory disease. BsAbs provide an off‐the‐shelf, repeatable immunotherapeutic approach, enabling rapid engagement of T cells against CD20‐positive MCL cells, often in the outpatient setting [16].

Both modalities are associated with characteristic toxicities. CRS is common with both BsAbs and CAR‐T, generally manageable with step‐up dosing, corticosteroids, and IL‐6 blockade. ICANS occurs more frequently with CAR‐T, particularly, CD28‐based products. Hematological toxicity, infections and prolonged cytopenias are important considerations, particularly in heavily pretreated patients. Careful patient assessment, bridging therapy, inpatient monitoring, and early recognition of adverse events are essential for optimizing safety [18].

Emerging evidence supports the rational sequencing of these therapies. BsAbs can achieve disease control and induce remission in patients not immediately eligible for CAR‐T and may be used in those relapsing after CAR‐T, although prior CAR‐T exposure can affect T‐cell fitness [43, 44]. Conversely, CAR‐T therapy remains highly effective following BsAb treatment, particularly in aggressive or high‐risk disease. Future strategies may include CAR‐T consolidation after BsAb‐induced remission or the use of BsAbs for relapse post‐CAR‐T, guided by patient fitness, disease kinetics, and prior therapy. Combination approaches—such as BsAbs with BTK inhibitors or CAR‐T with ibrutinib/acalabrutinib—are under investigation to enhance efficacy, persistence, and depth of response.

In summary, BsAbs and CAR‐T therapies represent complementary tools in the MCL treatment armamentarium. CAR‐T provides durable remissions and the potential for long‐term disease control, whereas BsAbs offer accessible, flexible therapy suitable for outpatient administration [45]. Optimal sequencing and combinatorial strategies, individualized to disease biology and patient characteristics, represent the next frontier for improving outcomes in this historically challenging disease.

## Author Contributions

**Santino Caserta**, **Enrica Antonia Martino**, and **Massimo Gentile:** conceptualization. **Ernesto Vigna**, **Antonella Bruzzese**, and **Fortunato Morabito:** methodology. **Santino Caserta:** writing – original draft preparation. **Massimo Gentile** and **Fortunato Morabito:** writing – review and editing. All authors have read and agreed to the published version of the manuscript.

## Ethics Statement

The authors have nothing to report.

## Conflicts of Interest

The authors declare no conflicts of interest.

## Data Availability

Data sharing not applicable to this article as no datasets were generated or analysed during the current study.
